# Association between non-high-density lipoprotein cholesterol to high-density lipoprotein cholesterol ratio (NHHR), atherogenic index of plasma and acute ischemic stroke classified by CISS

**DOI:** 10.3389/fneur.2026.1865857

**Published:** 2026-07-23

**Authors:** Zhiwei He, Mengna Lv, Jiaying Luo, Qingwen Li, Zhaohui Zhang, Xuan Niu

**Affiliations:** 1 Department of Neurology, Renmin Hospital of Wuhan University, Wuhan, China; 2 Department of Anesthesiology, Renmin Hospital of Wuhan University, Wuhan, China

**Keywords:** AIP, CISS, LAA, mediation, NHHR, PAD

## Abstract

**Background:**

Dyslipidemia is a significant contributor to ischemic stroke. The non-high-density lipoprotein cholesterol to high-density lipoprotein cholesterol ratio (NHHR) and the atherogenic index of plasma (AIP) are emerging indicators of cardiovascular risk. However, the relationship between NHHR and AIP in the context of ischemic stroke subtypes, as classified by the Chinese ischemic stroke subclassification (CISS) system, warrants further investigation. Additionally, the roles of NHHR and AIP in relation to hypertension or diabetes within these ischemic stroke subtypes remain unclear. Therefore, this study aims to elucidate the connections among them.

**Methods:**

Patients diagnosed with acute ischemic stroke subtypes of large artery atherosclerosis (LAA) and penetrating artery disease (PAD) according to the CISS classification criteria from 2018 to 2022 at the Department of Neurology, Renmin Hospital of Wuhan University, were selected for analysis. We compared baseline demographic information, past medical history, and biochemical test results. Multivariable logistic regression analysis was employed to investigate the association between NHHR and AIP with acute ischemic stroke subtypes. Receiver operating characteristic (ROC) analysis was utilized to assess the diagnostic value of these factors for acute ischemic stroke subtypes. Mediation analyses were conducted to determine whether NHHR and AIP mediate the relationship between exposure variables (hypertension and diabetes) and outcomes (LAA and PAD subtypes).

**Results:**

Univariate analysis revealed significant differences between the acute ischemic stroke subtype group and the non-stroke group regarding NHHR, AIP, gender, age, total bilirubin, direct bilirubin, high-sensitivity C-reactive protein, lipoprotein (a), diabetes, hyperhomocysteinemia, and hypertension (*p* < 0.05). In the multivariable logistic regression analysis, after adjusting for potential confounders, NHHR was found to be significantly associated with both LAA subtype (odds ratio [OR] = 1.323, 95% confidence interval [CI]: 1.131–1.547, *p* < 0.001) and PAD subtype (OR = 1.195, 95% CI: 1.001–1.425, *p* = 0.048). AIP was significantly related to LAA subtype (OR = 2.045, 95% CI: 1.094–3.823, *p* = 0.025), while the association with PAD subtype (OR = 1.740, 95% CI: 0.857–3.531, *p* = 0.125) was not statistically significant. NHHR appears to play a marginal role in mediating the potential effects of hypertension or diabetes on LAA subtypes. Specifically, hypertension has a minor impact on LAA subtype mediated by NHHR, with an average causal mediation effect (ACME) of 0.007 and a proportion mediated (PM) of 3.0%. Meanwhile, diabetes has a relatively larger impact on LAA subtypes mediated by NHHR, with an ACME of 0.017 and a PM of 10.0%.

**Conclusion:**

Elevated NHHR is a significant risk factor for the occurrence of both LAA and PAD subtypes according to the CISS criteria, whereas AIP is solely associated with LAA subtypes. Additionally, increased levels of NHHR mediate the relationship between hypertension, diabetes, and LAA subtypes.

## 1 Introduction

Cerebrovascular accidents are the second leading cause of death worldwide and represent a significant public health concern. In 2016, the number of stroke patients globally was estimated at 80.1 million, with the loss of disability-adjusted life years attributable to stroke being approximately 116 million (1). By 2019, this figure had risen to over 100 million stroke patients worldwide, reflecting an 85% increase in prevalence since 1990, with ischemic strokes accounting for more than 80% of cases (2, 3). Furthermore, over the past 30 years, the global risk of stroke among individuals aged 25 and older has risen to 24% (4).

For acute ischemic stroke, researchers have proposed various classification methods based on the etiology of the disease (5). A reasonable etiological classification system is crucial for the treatment and prognosis of ischemic stroke. The Trial of Org 10,172 in Acute Stroke Treatment (TOAST) classification is the most widely used system, and its validity has been empirically demonstrated (6, 7). The Chinese Ischemic Stroke Subclassification (CISS) is an innovative system that shares similar stroke diagnostic categories with TOAST, but its classification criteria provide more detailed insights into stroke pathophysiology (7). According to the CISS subdivision criteria, ischemic stroke is divided into several specific subtypes: large artery atherosclerosis (LAA), which includes atherosclerosis of the aortic arch and intra- or extracranial large arteries; cardiogenic stroke; penetrating artery disease (PAD); other etiologies; and undetermined etiology (7, 8). An examination of the CISS and TOAST classification systems revealed a strong correlation, with CISS demonstrating higher reliability among different evaluators (5). In contrast, the TOAST classification exhibits only moderate inter-rater reliability, as approximately 40% of strokes are categorized as “established stroke” (6).

The atherogenic index of plasma (AIP) is defined as the logarithmic transformation of the ratio of triglycerides (TG) to high-density lipoprotein cholesterol (HDL-C). This index not only reflects the TG to HDL-C ratio but also indicates the size of lipoprotein particles, which can be more informative than simply high TG or low HDL-C levels (9, 10). Recent observations indicate that elevated AIP is significantly associated with an increased risk of ischemic stroke, as noted in the China Health and Retirement Longitudinal Study (CHARLS) (11, 12). Furthermore, AIP is significantly linked to an increased risk of major cardiovascular events and serves as an independent prognostic factor in patients with CAD; its association with stroke is particularly significant in individuals with prediabetes and diabetes (12, 13). Recent findings have demonstrated that the TG/HDL-C ratio itself is associated with arterial stiffness in subjects with prediabetes, exhibiting a stronger correlation than the triglyceride-glucose index (14). In a large cross-sectional study of adults, AIP was identified as an independent factor associated with elevated carotid-femoral pulse wave velocity, and a strong positive correlation between AIP and brachial-ankle pulse wave velocity has been observed in patients with essential hypertension (15, 16). Collectively, these observations suggest that the established link between AIP and major adverse cardiovascular events may be partially mediated through the acceleration of arterial stiffness and attendant endothelial compromise (14, 16).

In a prior study, researchers observed that non-traditional lipid markers, such as the non-high-density lipoprotein cholesterol to high-density lipoprotein cholesterol ratio (NHHR), serve as independent predictors of carotid plaque vulnerability and the extent of stenosis (17). Elevated NHHR is significantly associated with an increased risk of coronary artery disease, particularly in elderly, hyperlipidemic, and non-diabetic patients (18, 19). A follow-up study conducted in Xi’an, China, which included 1,659 patients with non-disabling ischemic events, demonstrated that elevated NHHR heightens the risk of stroke recurrence in elderly patients within one year (20). Additionally, increasing evidence suggests that NHHR is a strong predictor of impaired fasting glucose (21), diabetes (22, 23), chronic kidney disease (24, 25), intracranial atherosclerosis, carotid plaque formation, high-risk plaques (26, 27), and metabolic syndrome.

This underscores the prevalence and significance of NHHR and AIP as predictors across various populations and disease states. Therefore, the mediating role of these indicators in the relationship between chronic diseases such as hypertension or diabetes and ischemic stroke warrants further exploration.

The purpose of this study was to explore: (1) the characteristics of patients with LAA and PAD subtypes; (2) the mediating effects of NHHR and AIP in hypertension or diabetes and ischemic stroke subtypes.

## 2 Materials and methods

### 2.1 Patients and participants

The study involved patients with acute ischemic stroke who were admitted to the hospital in the Department of Neurology, Renmin Hospital of Wuhan University from 2018 to 2022. This research received approval from the Ethics Committee of Renmin Hospital of Wuhan University (No. WDRM2023-K068). Additionally, the study was reported in accordance with STROBE guidelines and conducted in compliance with the Declaration of Helsinki.

The inclusion criteria for this study were as follows: (1) participants aged 18 years or older; (2) patients diagnosed with acute ischemic stroke classified as either the LAA or PAD subtype through imaging examinations (evaluated independently by 2 or more experienced neurologists). The exclusion criteria included: (1) any history of allergic reactions to iodine preparations; chronic inflammatory conditions; malignant tumors; blood disorders; or severe liver and kidney diseases; (2) patients who underwent incomplete examinations or test within 24 h of admission.

### 2.2 Data collection and measurement

Patient demographic characteristics, imaging information, and medical history data were collected, including hypertension, diabetes, and hyperhomocysteinemia. Laboratory tests, including blood and lipid assessments, were performed within 24 h of admission. We calculated the AIP and NHHR using the following formulas: AIP = log_10_ (TG/HDL-C) and NHHR = (Tch - HDL-C)/HDL-C. In addition to lipid levels, we also collected high-sensitivity C-reactive protein (hs-CRP), aspartate aminotransferase (AST), alanine aminotransferase (ALT), total bilirubin (TBIL), direct bilirubin (DBIL), and total bile acid (TBA) levels from patients to investigate differences in the degree of inflammation, liver function and bile acid metabolism.

### 2.3 Statistical analysis

All statistical analyses were conducted using the R statistical package (version 3.5.3) and SPSS (version 27). A double tailed *p*-value of < 0.05 was considered to indicate statistical significance in all analyses. In descriptive statistics, continuous variables are expressed as means and standard deviations or medians and interquartile ranges, and categorical variables as proportions and percentages of the total. The χ^2^ test was used to compare categorical variables between groups. Continuous variables were first checked for normality. Whenever normal distribution criteria were met, one-way analysis of variance tests was performed, whereas when results were not normally distributed, Kruskal–Wallis H tests were performed.

Multivariate logistic regression analysis was performed to evaluate the association between chronic diseases and stroke subtypes. Mediation analyses were performed to examine whether lipid markers mediated the relationship between the exposure variable (hypertension and diabetes) and outcome (stroke subtypes). Thousand bootstraps were used in our analysis. The size of the indirect pathway effect, average direct effect, proportion of the mediating effect, and *p*-value for the mediating effect are all shown in the results.

## 3 Results

A total of 1,098 patients were included in the study, with an average age of 55.5 years, of which 58.3% were male. According to the CISS classification criteria, 326 patients were diagnosed with LAA, while 203 patients were diagnosed with PAD. The baseline characteristics of patients with LAA and PAD subtypes are presented in Table 1. Compared to the non-stroke group, both the LAA and PAD subgroups exhibited higher levels of AIP and NHHR (*p* < 0.001). Additionally, the LAA and PAD subgroups had elevated levels of DBIL, TBIL, Lpa, and hs-CRP (*p* < 0.001). Furthermore, the prevalence of diabetes, hypertension, and hyperhomocysteinemia was significantly higher in two subgroups (*p* < 0.001).

**Table 1:** Characteristics of study participants.

Characteristics	PAD	*p* value	Non-stroke	*p* value	LAA
	*N* = 203		*N* = 569		*N* = 326
Age	61 (53.5, 69.0)	< 0.001	50 (39.0, 60.0)	< 0.001	64 (53.3, 71.0)
Gender					
Male	135 (66.5%)	< 0.001	266 (46.7%)	< 0.001	239 (73.3%)
Female	68 (33.5%)		303 (53.3%)		87 (26.7%)
Diabetes		< 0.001		< 0.001	
No	148 (72.9%)		517 (90.9%)		233 (71.5%)
Yes	55 (27.1%)		52 (9.1%)		93 (28.5%)
Hyperhomocysteinemia		< 0.001		< 0.001	
No	128 (63.1%)		505 (88.8%)		215 (66.0%)
Yes	75 (36.9%)		64 (11.2%)		111 (34.0%)
Hypertension		< 0.001		< 0.001	
No	71 (35.0%)		449 (78.9%)		130 (39.9%)
Yes	132 (65.0%)		120 (21.1%)		196 (60.1%)
AST	19 (16, 24)	0.888	19 (16, 24)	0.391	20 (16, 24)
ALT	18 (13, 25)	0.531	17 (12, 26)	0.101	18 (13, 27)
DBIL	4.0 (3.22, 5.15)	< 0.001	3.2 (2.50, 4.40)	< 0.001	4.2 (3.10, 5.68)
TBIL	13.5 (10.34, 16.73)	< 0.001	11.5 (8.89, 15.18)	< 0.001	13.2 (10.10, 16.78)
TBA	4.11 (2.33, 7.54)	0.759	4.34 (2.52, 6.81)	0.152	3.89 (2.41, 6.70)
CRP	1.51 (0.50, 6.42)	< 0.001	0.47 (0.10, 1.74)	< 0.001	3.08 (0.61, 9.16)
Lpa	163 (81.3, 309.8)	< 0.001	110 (57.0, 247.0)	< 0.001	167 (85.3, 366.5)
NHHR	3.4 (2.5, 4.3)	< 0.001	2.8 (2.1, 3.7)	< 0.001	3.5 (2.7, 4.3)
AIP	0.19 (0.29)	< 0.001	0.08 (0.32)	< 0.001	0.19 (0.27)

**Figure 1:**
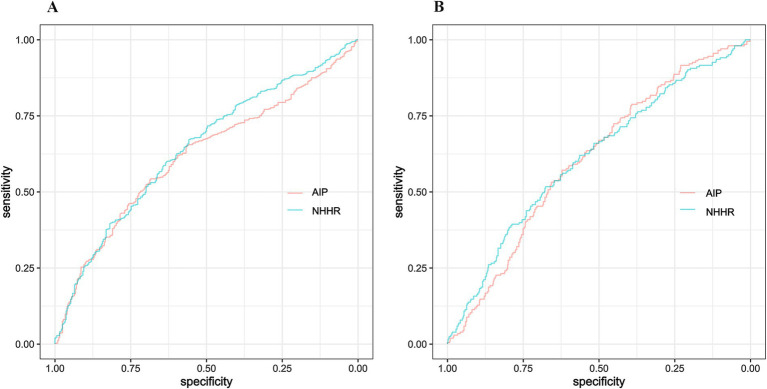
ROC curves of the NHHR and AIP for predicting LAA **(A)** and PAD **(B)** occurrence.

Receiver operating characteristic (ROC) analysis was conducted to evaluate the predictive ability of biomarkers for ischemic stroke (Figure 1). According to area under the curve (AUC), NHHR (LAA: AUC: 0.642, 95% confidence interval [CI]: 0.605–0.679; PAD: AUC: 0.614, 95%CI: 0.569–0.659) demonstrated a superior predictive capacity compared to AIP (LAA: AUC: 0.617, 95%CI: 0.580–0.654; PAD: AUC: 0.608, 95%CI: 0.565–0.651) in both the LAA and PAD subtypes. Table 2 presents the details regarding optimal cutoff points, specificity, and sensitivity. In the multivariable logistic regression analysis, after adjusting for potential confounders, NHHR remained significantly associated with the LAA subtype (odds ratio [OR] = 1.323, 95% CI: 1.131–1.547, *p* < 0.001) and the PAD subtype (OR = 1.195, 95% CI: 1.001–1.425, *p* = 0.048). Within both subtypes, individuals in the higher NHHR quartile exhibited a progressively increased risk when compared to those in the lowest NHHR quartile. Furthermore, NHHR was significantly positively correlated with both the LAA subtype (P for trend < 0.001) and the PAD subtype (P for trend = 0.022). Conversely, AIP was significantly associated with the LAA subtype (OR = 2.045, 95% CI: 1.094–3.823, *p* = 0.025) but did not maintain a significant relationship with the PAD subtype (OR = 1.740, 95% CI: 0.857–3.531, *p* = 0.125) (Table 3).

**Table 2:** AUC in predicting LAA and PAD occurrence.

Variables	AUC (95% CI)	Cutoff point	Sensitivity	Specificity
AUC in predicting LAA occurrence				
NHHR	0.642 (0.605–0.679)	3.09	0.635	0.596
AIP	0.617 (0.580–0.654)	0.77	0.687	0.54
AUC in predicting PAD occurrence				
NHHR	0.614 (0.569–0.659)	3.36	0.517	0.677
AIP	0.608 (0.565–0.651)	0.14	0.571	0.622

**Table 3:** Logistic regression models for LAA and PAD.

Subtypes	Categories	Model 1		Model 2		Model 3	
		HR (95% CI)	*p* value	HR (95% CI)	*p* value	HR (95% CI)	*p* value
LAA	NHHR	1.483 (1.317, 1.671)	<0.001	1.324 (1.158, 1.514)	<0.001	1.323 (1.131, 1.547)	<0.001
	Q1	Ref.		Ref.		Ref.	
	Q2	1.598 (1.042, 2.451)	0.032	1.137 (0.694, 1.863)	0.611	1.095 (0.638, 1.881)	0.742
	Q3	2.750 (1.817, 4.163)	<0.001	2.167 (1.344, 3.494)	0.002	2.249 (1.319, 3.836)	0.003
	Q4	3.835 (2.537, 5.797)	<0.001	2.523 (1.572, 4.048)	<0.001	2.609 (1.517, 4.487)	<0.001
	P for trend		—		—		<0.001
	AIP	3.247 (2.043, 5.159)	<0.001	2.304 (1.350, 3.933)	0.002	2.045 (1.094, 3.823)	0.025
	Q1	Ref.		Ref.		Ref.	
	Q2	1.908 (1.248, 2.919)	0.003	1.317 (0.803, 2.159)	0.275	1.068 (0.621, 1.837)	0.811
	Q3	3.602 (2.380, 5.452)	<0.001	2.462 (1.527, 3.969)	<0.001	2.427 (1.437, 4.100)	<0.001
	Q4	2.744 (1.808, 4.165)	<0.001	2.026 (1.263, 3.249)	0.003	1.740 (1.011, 2.993)	0.045
	P for trend		—		—		0.008
PAD	NHHR	1.379 (1.210, 1.571)	<0.001	1.290 (1.117, 1.489)	<0.001	1.195 (1.001, 1.425)	0.048
	Q1	Ref.		Ref.		Ref.	
	Q2	1.390 (0.839, 2.304)	0.202	1.141 (0.652, 1.998)	0.644	1.020 (0.558, 1.866)	0.948
	Q3	1.695 (1.034, 2.779)	0.036	1.423 (0.828, 2.446)	0.202	1.175 (0.641, 2.152)	0.603
	Q4	3.218 (2.006, 5.163)	<0.001	2.397 (1.420, 4.047)	0.001	1.886 (1.019, 3.489)	0.043
	P for trend		—		—		0.022
	AIP	3.000 (1.781, 5.051)	<0.001	2.608 (1.442, 4.715)	0.002	1.740 (0.857, 3.531)	0.125
	Q1	Ref.		Ref.		Ref.	
	Q2	1.652 (0.989, 2.759)	0.055	1.494 (0.846, 2.638)	0.166	1.057 (0.569, 1.963)	0.861
	Q3	2.759 (1.689, 4.507)	<0.001	2.102 (1.217, 3.632)	0.008	1.361 (0.738, 2.507)	0.323
	Q4	2.633 (1.610, 4.307)	<0.001	2.302 (1.334, 3.971)	0.003	1.606 (0.860, 3.000)	0.137
	P for trend		—		—		0.082

Model 1: unadjusted; Model 2: ajusted for age and gender; Model 3: ajusted for age, gender, hypertension, hyperhomocysteinemia, DM, DBIL, TBIL, Lpa, hs-CRP.

**Figure 2:**
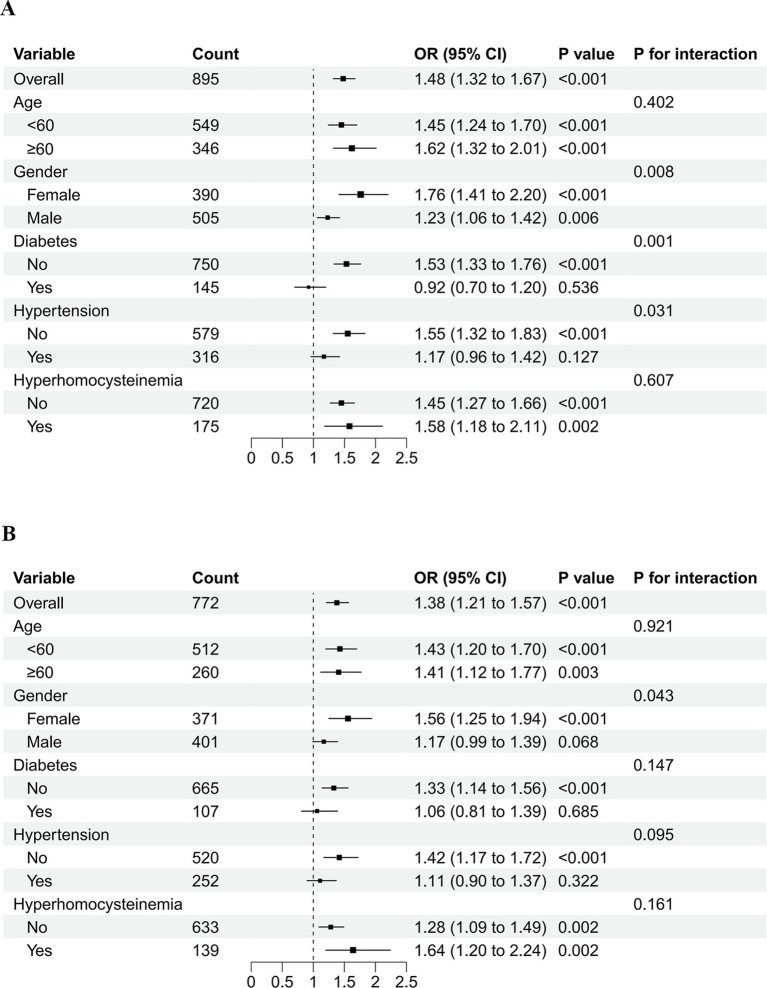
Subgroup analyses of the association between NHHR and stroke subtypes in the entire cohort: **(A)** LAA; **(B)** PAD.

**Figure 3:**
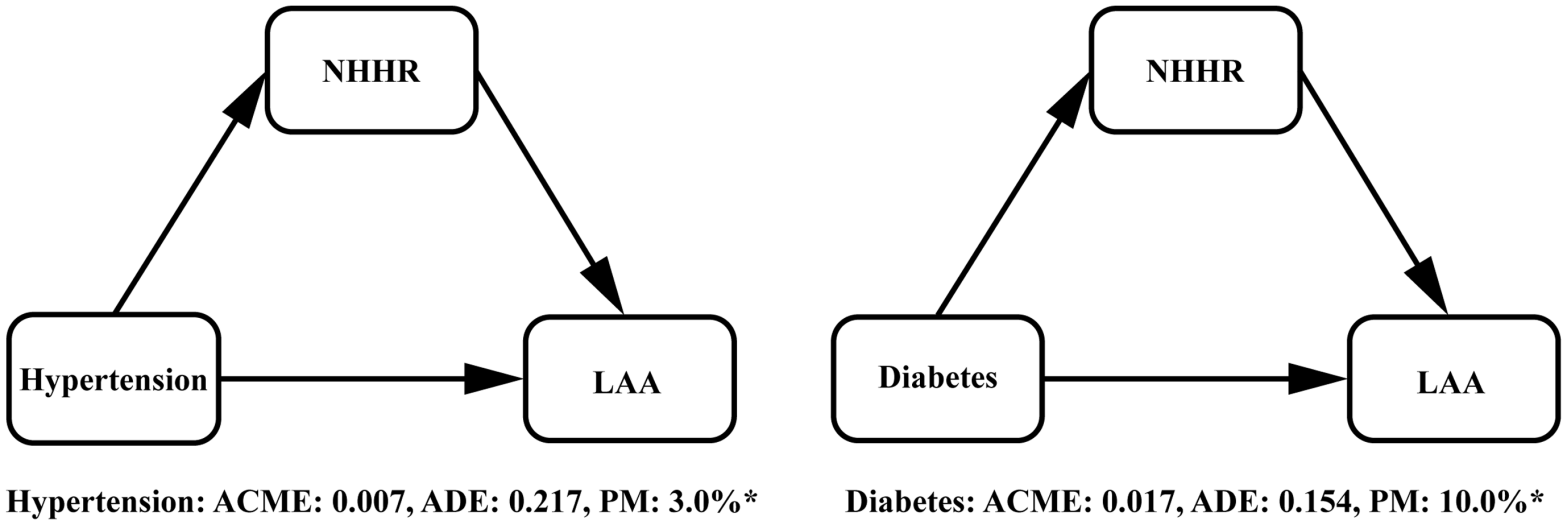
Mediational models. Figure demonstrating mediation models with independent variables of hypertension or DM, mediators of lipid markers and dependent variable of LAA. ACME indicates average causal mediation effects (e.g., indirect effect), ADE indicates average direct effects, and PM indicates proportion mediated effects, e.g., proportion of the total effect due to the mediator. ACME and ADE are displayed along with the PM. *Indicates *p* < 0.05.

In a subgroup analysis stratified by age, sex, hypertension, diabetes, and hyperhomocysteinemia, Figure 2 examines the association between NHHR and both stroke subtypes. For LAA subtypes, the interactions between NHHR and age, as well as hyperhomocysteinemia, were not significant. However, significant differences were observed in the interaction relationships between gender, hypertension, diabetes, and NHHR, with the following *p* values for interaction: gender: 0.008; hypertension: 0.031; diabetes: 0.001. In contrast, among PAD subtypes, only the interaction between NHHR and gender was significant (P for interaction = 0.043, P for interaction = 0.006). These findings suggest that NHHR is not only an independent predictor but also interacts with other factors to influence the occurrence of ischemic stroke. Besides, in exploring the mediating effect of lipid indicators on the relationship between hypertension or diabetes and the two subtypes, we found that NHHR mediates the association between hypertension or diabetes and LAA subtypes (hypertension: average causal mediation effects [ACME]: 0.007, proportion mediated [PM]: 3.0%, *p* = 0.046; diabetes: ACME: 0.017, PM: 10.0%, *p* = 0.016) (Table 4; Figure 3).

**Table 4:** Lipid markers mediating the association between hypertension or diabetes and stroke subtypes.

Exposure	Variables	AMCE				ADE				Total effect				Proportion mediated			
		Estimate	95% CI lower	95% CI upper	*p* value	Estimate	95% CI lower	95% CI upper	*p* value	Estimate	95% CI lower	95% CI upper	*p* value	Estimate	95% CI lower	95% CI upper	*p* value
Hypertension	NHHR																
	Unadjusted analyses	0.028	0.010	0.044	<0.001	0.368	0.310	0.436	<0.001	0.396	0.340	0.459	<0.001	0.071	0.035	0.120	<0.001
	Adjusted analyses	0.007	0.000	0.016	0.046	0.217	0.150	0.281	<0.001	0.224	0.160	0.287	<0.001	0.030	0.001	0.080	0.046
	AIP																
	Unadjusted analyses	0.018	0.010	0.031	0.002	0.395	0.300	0.493	<0.001	0.396	0.340	0.459	<0.001	0.046	0.018	0.080	0.002
	Adjusted analyses	0.003	0.000	0.010	0.28	0.221	0.150	0.284	<0.001	0.224	0.160	0.287	<0.001	0.012	−0.009	0.050	0.28
DM	NHHR																
	Unadjusted analyses	0.053	0.030	0.080	<0.001	0.278	0.190	0.366	<0.001	0.331	0.250	0.416	<0.001	0.160	0.087	0.270	<0.001
	Adjusted analyses	0.017	0.000	0.033	0.016	0.154	0.070	0.235	<0.001	0.171	0.090	0.253	<0.001	0.100	0.018	0.250	0.016
	AIP																
	Unadjusted analyses	0.036	0.020	0.059	<0.001	0.294	0.210	0.380	<0.001	0.331	0.250	0.416	<0.001	0.110	0.049	0.190	<0.001
	Adjusted analyses	0.007	−0.010	0.023	0.27	0.164	0.080	0.245	<0.001	0.171	0.090	0.253	<0.001	0.042	−0.045	0.170	0.27
Hypertension	NHHR																
	Unadjusted analyses	0.018	0.010	0.032	<0.001	0.370	0.300	0.441	<0.001	0.387	0.320	0.456	<0.001	0.046	0.014	0.090	<0.001
	Adjusted analyses	0.005	0.000	0.014	0.17	0.244	0.170	0.313	<0.001	0.249	0.180	0.317	<0.001	0.020	−0.010	0.060	0.17
	AIP																
	Unadjusted analyses	0.013	0.000	0.027	0.022	0.374	0.310	0.444	<0.001	0.387	0.320	0.456	<0.001	0.034	0.005	0.070	0.022
	Adjusted analyses	0.003	−0.010	0.012	0.43	0.246	0.170	0.315	<0.001	0.249	0.180	0.317	<0.001	0.012	−0.022	0.050	0.43
DM	NHHR																
	Unadjusted analyses	0.044	0.020	0.073	0.002	0.248	0.140	0.353	<0.001	0.292	0.190	0.388	<0.001	0.150	0.049	0.290	0.002
	Adjusted analyses	0.016	−0.010	0.040	0.164	0.146	0.050	0.240	0.002	0.162	0.070	0.255	<0.001	0.099	−0.050	0.320	0.164
	AIP																
	Unadjusted analyses	0.032	0.010	0.057	0.012	0.260	0.160	0.367	<0.001	0.292	0.190	0.388	<0.001	0.109	0.028	0.230	0.012
	Adjusted analyses	0.006	−0.010	0.025	0.428	0.156	0.060	0.247	0.002	0.162	0.070	0.255	<0.001	0.040	−0.092	0.190	0.428

Adjusted analyses adjusted for age, gender, hypertension, diabetes, hyperhomocysteinemia, TBIL, DBIL, Lpa, hs-CRP.

## 4 Discussion

Ischemic stroke is a prevalent condition affecting the nervous system. In this study, we conducted the first comprehensive analysis of the association between LAA and PAD subtypes within the CISS classification, as well as their relationship with the NHHR and AIP. Furthermore, we explored for the first time the mediating effects of NHHR and AIP between hypertension or diabetes and ischemic stroke.

Our findings indicate that elevated NHHR is associated with the occurrence of LAA and PAD ischemic stroke, while elevated AIP is linked to LAA subtypes. Previous studies have demonstrated that abnormal lipid levels significantly increase the risk of ischemic stroke (28). Recent clinical investigations have further elucidated the specific associations between these lipid indices and atherosclerotic stroke subtypes. Zhong et al. observed that elevated AIP was significantly associated with an increased risk of LAA, with each one-unit increment in AIP conferring 42% higher odds of LAA (29). In a multicenter cohort of LAA stroke patients undergoing endovascular treatment, Sohn et al. reported that the highest AIP quartile independently predicted futile reperfusionand early neurological deterioration (30). Besides, Kong et al. demonstrated that admission NHHR was independently associated with unfavorable 90-day functional outcomes in acute ischemic stroke (31). Collectively, our findings are consistent with these prior observations, and these observations support the notion that NHHR and AIP may serve as practical biomarkers for identifying atherosclerotic burden in specific ischemic stroke subtypes.

Non-HDL, a component of lipoproteins containing apolipoprotein B, serves as a more effective indicator of atherosclerosis compared to LDL (32, 33). Research has shown that elevated non-HDL cholesterol levels are closely associated with the formation of vulnerable plaques and endothelial damage. This underscores the importance of lowering non-HDL cholesterol levels in the primary prevention of stroke (34). From another perspective, HDL plays a critical role in reverse cholesterol transport, facilitating the return of cholesterol to the liver via class B type I scavenger receptors and preventing tumor necrosis factor alpha-induced apoptosis in endothelial cells. Abnormalities in HDL impair its ability to receive cholesterol through ATP-binding cassette transporter A1 and ATP-binding cassette transporter G1, while simultaneously activating inflammatory responses through the dysregulation of toll-like receptor 4 and nuclear factor kappa-B signaling in macrophages (35, 36). Lipid overload not only accelerates the development of atherosclerotic plaques but also exacerbates local inflammation and oxidative stress, further compromising endothelial function and plaque stability (37). Elevated TG levels are frequently linked to the formation of small, dense low-density lipoprotein particles, which are highly atherogenic. TG-rich lipoproteins and their remnants can activate endothelial cells and recruit inflammatory cells to the vascular wall. These processes contribute to local inflammation, increase the necrotic core’s content, and thin the fibrous cap, thereby diminishing the stability of atherosclerotic plaques and rendering them more susceptible to rupture and subsequent stroke (38, 39).

Our study demonstrated that NHHR and AIP were more closely associated with LAA subtypes than with PAD subtypes. Previous research has indicated that LAA and PAD subtypes exhibit distinct differences in their pathological underpinnings. The blood vessels primarily responsible for the development of LAA subtypes predominantly include the intracranial and extracranial large arteries, such as the aortic arch. Following the onset of atherosclerosis in these large arteries, pathological processes—such as plaque formation, vascular stenosis, or occlusion—facilitate the occurrence of stroke (7). Traditional risk factors, including dyslipidemia, hypertension, and diabetes, contribute significantly to the incidence of ischemic stroke (40). The PAD subtype specifically addresses lesions of the perforator artery itself and its associated factors. Cerebral perforating arteries are small terminal blood vessels that supply blood to deep brain structures. Owing to their diminutive size and significant perfusion requirements, these vessels are particularly vulnerable to damage resulting from hypertension. Besides, atherosclerosis occurring proximal to the perforating arteries or lipid hyalinization within small arteries can lead to these lesions, resulting in inadequate blood supply to the perforating arteries and subsequently causing stroke (41).

It should be noted that NHHR and AIP demonstrated only modest discriminative ability as individual biomarkers for LAA and PAD in the present study. A study by Wang et al. reported that AIP demonstrated favorable predictive value for early neurological deterioration in patients with acute ischemic stroke (AUC = 0.681), and Liao et al. further demonstrated that the combined model of AIP and NHHR achieved good accuracy in predicting early neurological deterioration after thrombolysis (AUC = 0.795) (42, 43). This inherent dual-characteristic suggests that their clinical utility may be substantially enhanced when incorporated into multivariable predictive models rather than applied in isolation. Consequently, their clinical utility may be substantially enhanced when incorporated into multivariable predictive models rather than used as standalone measures.

Our findings suggest that NHHR moderately mediates the association between diabetes or hypertension and LAA subtypes. Numerous studies have indicated that patients with diabetes or hypertension frequently exhibit abnormal lipid levels (44–46). In a study derived from the China Health and Retirement Longitudinal Study (CHARLS), an elevated NHHR was identified as a significant risk factor for cardiovascular disease among middle-aged and older Chinese adults, with this association being more pronounced in individuals younger than 60 years and in those with hypertension (47). Similarly, Li et al. reported that a higher atherogenic index of plasma was significantly associated with incident hypertension and type 2 diabetes mellitus in a longitudinal follow-up study (48). Zeng et al. observed that individuals with both elevated triglyceride-glucose index and AIP had a higher hazard ratio for cardiovascular disease (49). Additionally, Wu et al. noted that patients with coronary artery disease and concurrent hypertension or diabetes exhibited significantly higher AIP levels and carotid atherosclerotic burden compared with those without these comorbidities (50). Lipid deposition diminishes the activity of endothelial cell nitric oxide synthase and reduces the bioavailability of nitric oxide, thereby increasing sensitivity to vasoconstrictors such as endothelin-1 and thromboxane A2, which are potent vasoconstrictors that further exacerbate the tendency for vasoconstriction, elevate systemic vascular resistance, and contribute significantly to the development of hypertension (51, 52). Vascular damage, oxidative stress, inflammation, and dysregulation of the renin-angiotensin system also play a contributory role (53). Elevated lipids exacerbate insulin resistance by directly affecting tissues such as the liver, muscle, and adipose tissue (54). The inflammatory response triggered by lipid accumulation further aggravates insulin resistance. Conversely, diabetes negatively impacts lipid metabolism, creating a vicious cycle in which insulin resistance and lipid dysregulation mutually reinforce each other (55). Consequently, these observations suggest that elevated NHHR associated with hypertension or diabetes may represent a partial associative link between chronic diseases and ischemic stroke.

However, within the context of this study, we have not yet observed that AIP mediates the relationship between hypertension or diabetes and stroke subtypes. This may imply that the effect of AIP could be influenced by other variables not examined in this study, such as genetic background, environmental factors, medication status, or individual variations in the progression of specific chronic diseases. In future research, we plan to expand the study sample size to more accurately elucidate the relationship between these factors.

Cardiovascular risk in diabetic patients represents a complex pathological process driven by multiple factors, encompassing residual lipid risk, residual inflammatory risk, endothelial dysfunction, and metabolic reprogramming (56). Emerging evidence indicates that hyperglycemia accelerates atherosclerosis through macrophage inflammatory reprogramming, endothelial metabolic switching, and oxidative stress-mediated vascular injury, collectively promoting plaque vulnerability (57). Furthermore, diabetic patients frequently exhibit atherogenic dyslipidemia characterized by elevated triglycerides, reduced HDL-C, and increased small dense LDL particles, with substantial residual cardiovascular risk persisting despite intensive LDL-C reduction (58, 59). Recent clinical investigations have demonstrated that SCORE2-Diabetes exhibits superior discriminative capacity for subclinical vascular damage compared to SCORE2, as reflected by stronger correlations with pulse wave velocity, intima-media thickness, and carotid atherosclerosis in patients with type 2 diabetes (60, 61). Concurrently, atherogenic lipid indices including AIP and NHHR have emerged as independent predictors of vascular complications in T2D. Our study observes that NHHR and AIP may mediate the relationship between diabetes and acute ischemic stroke; integrating these lipid indices with SCORE2-Diabetes may offer a more refined framework for cardiovascular risk stratification in diabetic subjects, thereby facilitating earlier identification of individuals at heightened risk for cerebrovascular events.

Our study has several limitations. First, the retrospective design of this study precludes the establishment of causal relationships between NHHR or AIP and the two stroke subtypes. Second, as with all observational studies, there may be potential uncontrolled or unmeasured confounders (such as menopausal status, the use of lipid-lowering drugs, or dietary habits) that could influence the study results, despite adjustments for known confounders. Furthermore, lipid parameters were measured within 24 h of admission following acute ischemic stroke. We cannot entirely exclude the possibility that acute stress responses, acute-phase reactants, or prestroke statin use may have influenced the measured values. Consequently, whether NHHR and AIP precisely reflect pre-stroke vascular risk profiles or represent post-stroke metabolic alterations remains uncertain. Finally, the participants in this study were drawn from a single-center cohort, which may limit the generalizability of the findings to other populations.

As concerns strengths, our study presents a comprehensive analysis of the association between lipid markers and stroke subtypes. It further investigates the mediating effects of NHHR and AIP in the occurrence of ischemic stroke associated with hypertension and diabetes. We utilized NHHR and AIP levels as lipid markers due to their cost-effectiveness and ease of clinical application.

## 5 Conclusion

The results of this study suggest a potential association between NHHR and AIP with ischemic stroke classified according to the CISS criteria. Notably, the effects of hypertension and diabetes on ischemic stroke are partially mediated by NHHR. These findings may highlight the importance of monitoring elevated NHHR and AIP levels, potentially supporting their management in the primary prevention of ischemic stroke. Further longitudinal studies are warranted to clarify whether patients with ischemic stroke are particularly susceptible to high NHHR driven by diabetes or hypertension, which could inform future treatment strategies.

## Data Availability

The raw data supporting the conclusions of this article will be made available by the authors, without undue reservation.

## Glossary

NHHR: non-high-density lipoprotein cholesterol to high-density lipoprotein cholesterol ratio
AIP: atherogenic index of plasma
CISS: Chinese ischemic stroke subclassification
PAD: penetrating artery disease
LAA: large artery atherosclerosis
TOAST: Trial of Org 10172 in Acute Stroke Treatment
hs-CRP: high-sensitivity C-reactive protein
TBIL: total bilirubin
DBIL: direct bilirubin
Lpa: Lipoprotein (a)
AST: aspartate aminotransferase
ALT: alanine aminotransferase
TBA: total bile acid
HDL-C: high-density lipoprotein cholesterol
TG: triglycerides
OR: odds ratio
CI: confidence interval
ACME: average causal mediation effect
ADE: average direct effect
PM: proportion mediated
CHARLS: China Health and Retirement Longitudinal Study
ROC: Receiver operating characteristic
AUC: area under the curve
